# Supplementary material for: Empirical methods for controlling false positives and estimating confidence in ChIP-Seq peaks
Source: BMC Bioinformatics. 2008 Dec 5;9:523. doi: 10.1186/1471-2105-9-523 (PMC2628906; doi:10.1186/1471-2105-9-523)
Supplement: Additional File 1 — A variety of html documents from the USeq web site detailing the available applications, their best usage, output file type descriptions, command line menus, etc. [file 1471-2105-9-523-S1.zip › USeqUserGuides/JavaDoc.zip!/JavaDoc/index-files/index-16.html]

Q-Index


|  |  |  |
| --- | --- | --- |
| **Overview** \| Package \| Class \| Use \| **Tree** \| **Deprecated** \| **Index** \| **Help** | |  |
| **PREV LETTER**   **NEXT LETTER** | **FRAMES**    **NO FRAMES**     **All Classes** |  |


A B C D E F G H I J L M N O P Q R S T U V W X Z 

---


## **Q**

**QCSeqs** - Class in edu.utah.seq.analysis: Takes PointData and generates sliding window average scores. **QCSeqs(String[])** - Constructor for class edu.utah.seq.analysis.QCSeqs: **QualityFileParser** - Class in util.bio.parsers: This class will read in combine quality files associated with raw sequencing results, skips blank lines, parses off the > character. **QualityFileParser(String)** - Constructor for class util.bio.parsers.QualityFileParser: **quantifyItems()** - Method in class util.bio.parsers.gff.GffParser: **Quantile** - Class in trans.cel: Class holding info relating to one quantile value. **Quantile(int, float)** - Constructor for class trans.cel.Quantile: **QuantileNormalization** - Class in trans.cel: Performs quantile normalization on arrays of double values. **QuantileNormalization()** - Constructor for class trans.cel.QuantileNormalization: **QuantileNormalization(File[])** - Constructor for class trans.cel.QuantileNormalization: **quantileNormalize()** - Method in class trans.cel.QuantileNormalization: Performs a median scaling then quantile normalization of the loaded float[replicas][intensities]. **quartiles(float[])** - Static method in class util.gen.Num: Calculates the Q1,Q2,and Q3 (25th, 50th, and 75th percentile). **queryOrder()** - Method in class util.apps.Flash: **queryShuffle()** - Method in class util.apps.Flash: **QVALUE\_ID** - Static variable in class edu.utah.seq.analysis.ScanSeqs

---


|  |  |  |
| --- | --- | --- |
| **Overview** \| Package \| Class \| Use \| **Tree** \| **Deprecated** \| **Index** \| **Help** | |  |
| **PREV LETTER**   **NEXT LETTER** | **FRAMES**    **NO FRAMES**     **All Classes** |  |


A B C D E F G H I J L M N O P Q R S T U V W X Z 

---
